# MASMDDI: multi-layer adaptive soft-mask graph neural network for drug-drug interaction prediction

**DOI:** 10.3389/fphar.2024.1369403

**Published:** 2024-05-20

**Authors:** Junpeng Lin, Binsheng Hong, Zhongqi Cai, Ping Lu, Kaibiao Lin

**Affiliations:** ^1^ School of Computer and Information Engineering, Xiamen University of Technology, Xiamen, China; ^2^ School of Economics and Management, Xiamen University of Technology, Xiamen, China

**Keywords:** drug-drug interactions, substructure interactions, co-attention, graph structure learning, molecular graph

## Abstract

Accurately predicting Drug-Drug Interaction (DDI) is a critical and challenging aspect of the drug discovery process, particularly in preventing adverse reactions in patients undergoing combination therapy. However, current DDI prediction methods often overlook the interaction information between chemical substructures of drugs, focusing solely on the interaction information between drugs and failing to capture sufficient chemical substructure details. To address this limitation, we introduce a novel DDI prediction method: Multi-layer Adaptive Soft Mask Graph Neural Network (MASMDDI). Specifically, we first design a multi-layer adaptive soft mask graph neural network to extract substructures from molecular graphs. Second, we employ an attention mechanism to mine substructure feature information and update latent features. In this process, to optimize the final feature representation, we decompose drug-drug interactions into pairwise interaction correlations between the core substructures of each drug. Third, we use these features to predict the interaction probabilities of DDI tuples and evaluate the model using real-world datasets. Experimental results demonstrate that the proposed model outperforms state-of-the-art methods in DDI prediction. Furthermore, MASMDDI exhibits excellent performance in predicting DDIs of unknown drugs in two tasks that are more aligned with real-world scenarios. In particular, in the transductive scenario using the DrugBank dataset, the ACC and AUROC and AUPRC scores of MASMDDI are 0.9596, 0.9903, and 0.9894, which are 2% higher than the best performing baseline.

## 1 Introduction

Given the limited clinical efficacy of a single drug in disease treatment, the management of complex diseases in humans often necessitates the concurrent use of multiple drugs (Horikawa and Sugimoto, 2019). Nevertheless, the concurrent administration of two or more drugs can give rise to Drug-Drug Interaction (DDI), wherein the chemical and physical interactions between drugs result in synergistic or antagonistic effects (Ryu et al., 2018). The intricate nature of drug interactions and their potential adverse effects in clinical settings continues to be a pivotal concern for healthcare professionals and researchers alike. Adverse effects resulting from these interactions not only compromise the effectiveness of the treatment, but also pose a significant threat to the patient’s health and life (Giacomini et al., 2007). In addition, the identification of DDIs is a critical determinant in drug safety evaluations and sometimes leads to the withdrawal of drugs from the market, underscoring the urgency of understanding and preventing such events (Tatonetti et al., 2012). Therefore, to avoid the harm caused by DDIs, the detection of interactions between co-administered drugs has always been a concern for biologists, pharmacologists, and clinicians. Given the complexity and multitude of potential drug combinations, relying solely on conventional approaches like *in vitro* experiments and clinical trials for DDI detection proves impractical due to their time-consuming, inefficient, and costly nature (Whitebread et al., 2005). In response to these limitations, recent years have witnessed a significant shift in research focus towards computer-based computational methods as a promising avenue for DDI prediction (Nyamabo et al., 2021). These methods employ techniques such as deep learning to learn from existing drug interaction data and construct models for predicting DDIs. In particular, the use of these methods offers a remarkable advantage in terms of speed and cost-effectiveness, revolutionizing the landscape of DDI prediction and mitigation strategies (Gottlieb et al., 2012; Tatonetti et al., 2012).

Recent advances in the field have resulted in significant contributions, with the main approaches being deep learning based (Ryu et al., 2018; Liu et al., 2019; Yan et al., 2019; Liu et al., 2022). So far, there are mainly three types of DDI prediction methods: similarity-based methods, network-based methods, and matrix factorization-based methods (Yan et al., 2019). Similarity-based methods are the predominant approach. They operate under the assumption that drugs sharing similar feature information are more inclined to exhibit comparable interactions. For instance, Vilar et al. (2012) identified new DDIs based on the similarity of drug structures in known DDI data. Network-based methods deduce probable DDIs by constructing different biologically relevant networks and acquiring the embedded representation of drug nodes in the network. More recently, Kang et al. (2022) proposed a deep fusion method for predicting drug-drug interactions based on drug features and topological relationships. Furthermore, matrix factorization-based methods decompose the adjacency matrix of DDIs into several factor matrices and reconstruct the adjacency matrix to identify potential DDIs (Narita et al., 2012; Wang et al., 2015). For example, Rohani et al. (2020) combined nonlinear multi-similarity fusion with matrix factorization for DDI prediction.

While the learning capabilities of the above deep learning methods have been shown to be efficient, no consideration has been given to extracting features from the original characterization of the drug (e.g., chemical structure), which is still new room for exploration for the DDI prediction task. Based on pharmaceutical chemistry knowledge, drugs are entities composed of various functional groups or chemical substructures. These functional groups or chemical substructures dictate all pharmacokinetic and pharmacodynamic properties of a drug and ultimately determine all its interactions (Harrold and Zavod, 2014). Therefore, recent research has shifted focus to these substructures, recognizing that DDIs are influenced by the presence of specific substructures in drugs. This has led to innovative approaches, such as Huang et al.'s (Huang et al., 2020b) CASTER framework represents a notable effort in predicting DDIs by refining drug chemical structures into substructural components. The drawback of this type of approach is that it neglects to extract topological information from the molecular map. In contrast, the graph neural network (GNN) technique is able to directly take the molecular map as an input and is able to capture the complex interactions of atoms and bonds in the molecule through the information transfer between the nodes. This is clearly favorable for DDI prediction. An example is the SSI-DDI model proposed by Nyamabo et al. (2021). Their model used a graph-attention network layer to extract substructural information of drug molecules.

However, there are still shortcomings in extracting molecular graph information using GNN technology. One primary issue is that not all detailed structures in molecular graphs are relevant to the DDI prediction task. Traditional GNNs use fixed subgraph sizes or predefined subgraph structures to extract subgraph information, making it difficult to capture important subgraph structures. Additionally, molecular graphs also contain noise information and lack flexibility in dealing with complex graphs, which can negatively affect the accuracy of the final prediction. Therefore, the precise extraction of subgraph information, focusing on the key elements is crucial for DDI prediction. To overcome the challenges posed by irregularities in the sizes and shapes of substructures and enhance the prediction accuracy of DDIs, we propose a novel method named Multi-layer Adaptive Soft Mask Graph Neural Network (MASMDDI). MASMDDI integrates soft-mask graph neural networks and substructure attention mechanisms to facilitate full access to drug features and their interactions. By extracting substructures from molecular graph structures, MASMDDI ensures a more nuanced characterization and allows for more flexible extraction of the necessary subgraphs using soft masking mechanisms. Consequently, this methodology enhances the capability to differentiate task-relevant structural details during downstream processing tasks, with a particular emphasis on addressing long-range dependencies prevalent in the higher layers of deep models. Furthermore, we introduce attention mechanism to mine substructure feature data and update latent features, facilitating the incorporation of complex information into the model. The final feature representation is constructed by linking these updated features, and a common attention mechanism is utilized to determine importance weights by learning interaction scores between the core substructure features of two drugs. The use of the common attention mechanism enables the simultaneous consideration of correlations between multiple subgraphs, thus capturing the semantic information between them more efficiently.

The main contributions of this study are as follows.

(1) We propose a DDI prediction method, MASMDDI, based on a multi-layer adaptive soft mask map neural network, which mines molecular map substructure feature information and updates potential features for DDI prediction.
(2) In the process of substructure extraction, MASMDDI extracts substructure-related information from the original drug molecule maps and learns the sequence characterization of independent subgraphs through a multi-layer adaptive soft mask map neural network, which acquires the ability to learn the characterization efficiently.
(3) MASMDDI uses an attention mechanism to update latent feature data for substructure embedding and exploits the correlation between core chemical substructures to identify information about interacting substructures. This enhances the final feature representation of drugs and increases the predictive accuracy of DDIs.
(4) We conducted extensive experiments on real-world drug datasets to validate the superiority of our proposed model when competing with state-of-the-art baselines in DDI prediction tasks. It performs well in both transductive and inductive settings.

The remainder of the paper is organized as follows. Section 2 discusses the work related to DDI prediction. Section 3 introduces the methodology for implementing MASMDDI. Section 4 describes the experiments conducted in this paper. Section 5 summarizes the contents of this paper.

## 2 Related work

In this section, we will delve into the current research on DDI prediction tasks from two aspects: drug molecular representations and the DDI prediction task.

### 2.1 Drug molecular representation

The representation of drug molecules plays a crucial role in drug-related tasks. Simplified Molecular Input Line Entry System (SMILES), as the most commonly used molecular descriptor, is a string where each atom is represented by its respective ASCII symbol code, and chemical bonds, branches, and stereochemistry are indicated by specific symbols in the SMILES string. By utilizing a vertical-first traversal tree algorithm, SMILES sequences can convert complex chemical structures into a tree generating character sequence (Lin et al., 2023). Various deep learning models, such as recurrent neural networks, can leverage their internal states to handle variable-length input sequences. Using SMILES sequences as input, these models employ various natural language processing techniques to extract chemical context. Sequence-based representations are often concise, memory-efficient, and easy to search. In alignment with these advantages, our study capitalizes on the inherent strengths of SMILES sequences by further transforming them into graph structures to represent drugs. This strategic approach aims to encapsulate and preserve the intricate relationships between molecular entities by exploiting the wealth of structural information encoded in these graphs.

Some research methods are based on the hypothesis that similar drugs may have similar chemical activities (Vilar et al., 2012). These methods represent drugs as similarity vectors for further preprocessing, often employing similarity metrics such as cosine similarity, Jaccard similarity, etc., to indicate the degree of similarity to other drugs in the representation space. These representations are limited to current human knowledge and cannot flexibly discover information beyond domain expert knowledge. In recent years, deep learning models called GNN designed for graph structures have been applied to generalize chemical molecules, especially in the learnable task representation of drugs, improving performance in molecular-related tasks (Yang et al., 2019). Some recently proposed methods have started to consider the importance of functional groups/chemical substructures in DDI (Nyamabo et al., 2021; Nyamabo et al., 2022). However, noise is introduced at each GNN layer, and nodes fail to capture drug substructure representations effectively. Our study introduces a soft-mask GNN layer, free from the constraints of fixed samples or dropout rates, which can better capture task-related substructures and skip noisy portions.

### 2.2 DDI prediction task

With the continuous increase in data volume and the constant evolution of algorithms, deep learning has made significant breakthroughs in various fields, including its application in drug-related prediction tasks (Zeng et al., 2020; Yang et al., 2022). Initially, most research efforts focused on developing effective representation methods to extract hidden embeddings from various public datasets (Xu et al., 2017). In contrast to methods based on traditional machine learning, these approaches no longer heavily depend on manual features and domain knowledge; instead, they extract more abstract information through deep learning frameworks. The deep learning-based approach eliminates the need for manual selection and adjustment of features, and the learned latent embeddings are ultimately used for predicting downstream tasks.

DDI prediction models can be configured for different classification tasks to serve varying prediction goals. Common DDI prediction tasks include the DDI binary classification task, the DDI multiclassification task, and the DDI multilabel classification task. Binary classification tasks aim to predict the existence of interactions between drug pairs. Nowadays, there are a large number of models for DDI binary prediction. For example, Lin et al. (2020) proposed the KGNN model, which used the GNN technique to extract drug topological features from the drug knowledge graph for DDI prediction. Considering that there will be jump similarity between drug nodes, Huang et al. (2020a) constructed the SkipGNN model. The model constructed a drug jump graph and fed it into the model along with a DDI graph to learn the feature vectors of drugs using iterative fusion.

In contrast, multi-class classification tasks aim to predict and distinguish specific DDI types between drugs. Some well-known DDI multiclassification models include the DDIMDL model, designed by Deng et al. (2020). This model selected data from DrugBank to construct a 65-class DDI dataset, and then extracted drug feature information using deep neural networks. Lin et al. (2022) developed the MDF-SA-DDI model and conducted multiclassification experiments on the dataset provided by Deng et al. (2020) The model was trained on drug pairs in various combinations using an autoencoder to learn the embedding representation of drug pairs, resulting in improved performance. To further enhance the model’s prediction accuracy, Lin et al. (2022) proposed the MDDI-SCL model. The model presented a supervised comparative learning strategy, which improved its ability to distinguish and identify drug-drug interactions.

Furthermore, multi-label classification tasks involve predicting one or more DDI types that may exist for each pair of drugs. This task requires considering the possibilities of multiple drug interactions, providing more comprehensive information for integrated treatment plans and drug management. These different classification tasks allow customized modeling for different prediction goals and requirements, providing more precise guidance for decision-making and clinical practices in the pharmaceutical field. There are also some models attempting multi-label DDI prediction tasks. For example, Feng et al. (2022) designed the deepMDDI model, which was constructed as an encoder of a relational graph convolutional network and a tensor-like decoder for unified modelling of interactions. Moreover Han et al. (2023) designed the MCFF-MTDDI model, which extracted drug chemical structure features, additional labelling features of drug pairs, and knowledge graph (KG) features of drugs. On top of that, a multi-channel fusion module was designed to fuse this information effectively, which represented high performance on both multi-classification tasks and multi-labelling tasks.

This study belongs to the multi-class classification task, where the objective is to predict the specific types of DDIs for each drug pair. During the model training process, model parameters are optimized by minimizing the cross-entropy loss in the multi-class classification task. This loss function is employed to measure the dissimilarity between the predicted probability distribution and the true distribution of DDI classes. By optimizing these parameters, the model aims to make accurate predictions regarding the specific types of interactions between drugs. The focus on multi-class classification in this study holds significance, as it allows for a more granular understanding of the diverse landscape of DDIs. Rather than providing a binary outcome, this model endeavors to discern and classify the specific nature of interactions, contributing to a more nuanced and clinically relevant prediction. This approach not only enhances the precision of DDI predictions but also improves the overall generalization performance of the model, making it more robust and applicable to diverse scenarios in drug interaction analysis.

### 2.3 GNN-based DDI prediction method

In recent years, with the rise of graph neural network technology, an increasing number of people have realized the importance of graph data. Simultaneously, the application of GNN in the DDI prediction task is becoming more widespread. Typically, GNN-based DDI prediction methods can be divided into two categories: methods based on DDI graphs and methods based on drug molecular graphs.

The DDI graph-based approach considers drugs as nodes, connects the drugs that will undergo DDI into an edge, constructs a DDI graph, and learns the topological features of the drugs in that graph. The topological features of a drug map the potential link between two drugs that would undergo a DDI, so this type of approach works to learn more effective topological features of the drug. Zitnik et al. (2018) constructed a multi-relationship network for protein-protein interactions, drug-protein target interactions, and multiple drug side effects. They developed a novel graph convolutional neural network for predicting multiple relational links in multimodal networks. Wang et al. (2022) classified DDI graphs into DDI-increasing and DDI-decreasing graphs based on the type of DDI response and used GCN to extract the drug embedding vectors in both types of graphs. Although these methods have achieved good prediction performance, there are some challenges that need to be addressed. For example, if only the DDI graph is used as the model’s input, it is not possible to learn the drug embedding vectors of new drugs. This is because the topological relationship of the new drug is unknown. Therefore, this type of method faces difficulty in fulfilling the task of new drug prediction.

The drug molecular graph-based approach uses atoms as nodes and chemical bonds as edges to extract features of drug molecular graph using GNN technique. The use of GNN technology to extract information from drug molecular graphs has the following advantages: 1) GNNs can automatically learn useful features from molecular graphs, avoiding the need for traditional machine-learning based methods that require a lot of effort in feature engineering; 2) GNNs can adequately capture complex interactions and relationships between atoms in molecules by means of message passing, which is clearly advantageous for DDI prediction. At the same time this type of method can effectively solve the problem of new drug prediction because the molecular graphs of new drugs are usually known. The SSI-DDI model constructed by Nyamabo et al. (2021) is one of the classics in the field. The model utilized graph attention networks to update the features of nodes in molecular graphs. Additionally, a joint attention mechanism was introduced to enhance the performance and interpretability of the model. Moreover, He et al. (2022) proposed the MFFGNN model and designed a molecular graph feature extraction module for extracting both global features of molecular graphs and local features of each atom in the molecular graph. This enabled the model to better learn the topological information of drug molecular graphs.

## 3 Methodology

In this section we describe in detail the workflow and building block unit of the MASMDDI method, including its multi-layer structure, adaptive learning mechanism and the substructure attention module. Specifically, MASMDDI is centered on the use of graph neural networks to capture complex chemical information in the structure of a drug molecule, and we further utilize the adaptive learning mechanism to extract key chemical substructures. In more detail, six key subsections are included in this section, which are problem definition, input data, substructure extraction, potential feature update, substructure interaction correlation, and DDI prediction and loss function.

**FIGURE 1:**
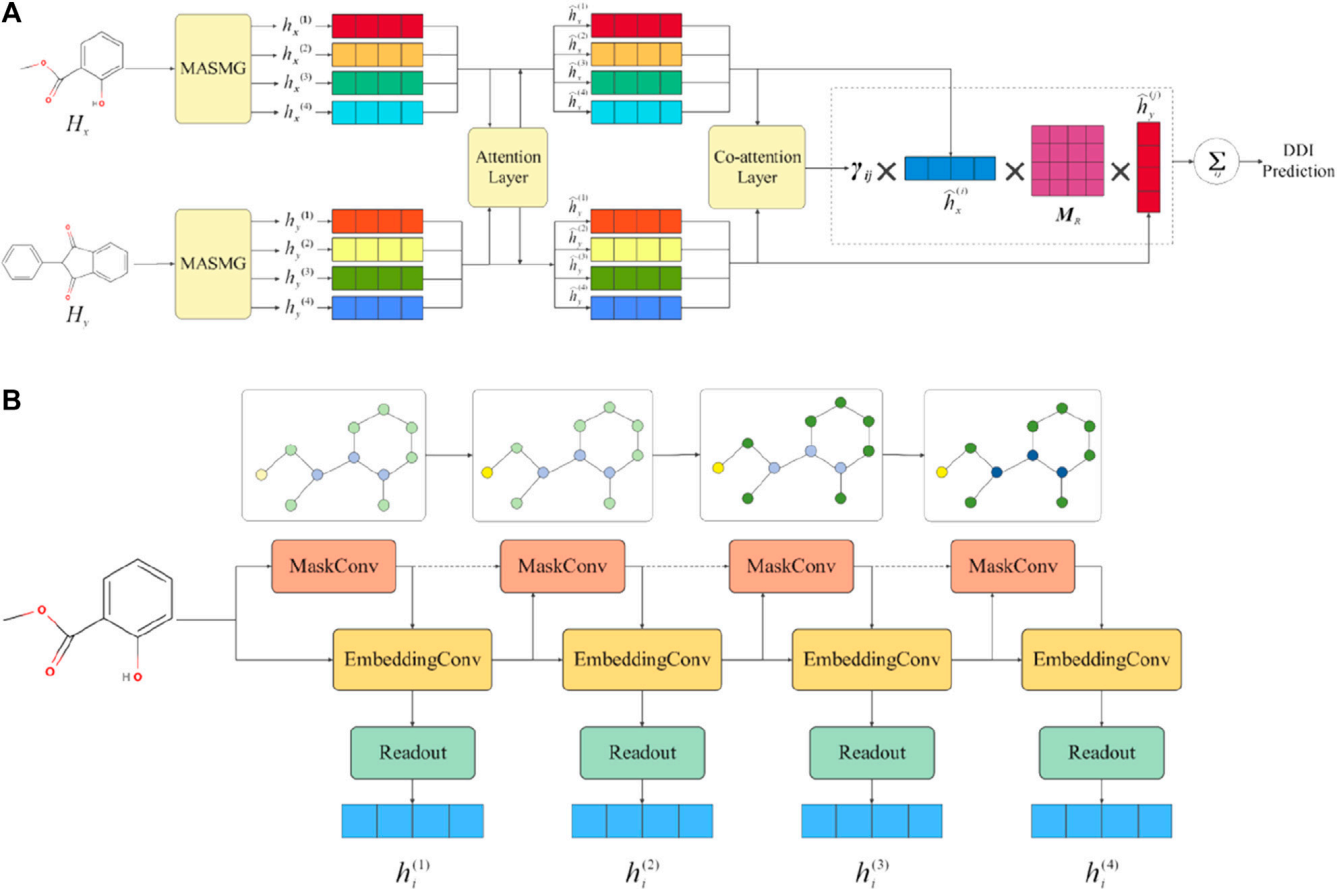
Overall framework diagram of MASMDDI model. Panel **(A)** presents an overview of the MASMDDI workflow. Panel **(B)** is the substructure extraction module MASMG, the gradual change in node color represents the process of subgraph learning.

Figure 1 shows the overall framework of the model. More specifically, Figure 1A describes the overall workflow framework of MASMDDI. The general workflow of this study involves the following procedural steps: 1) Given a DDI tuple, the graphical representation of the drug is taken as input. 2) Extract substructure information through the application of multiple layers of soft-mask graph neural network layers and readout operations. 3) Update latent features based on a substructure attention mechanism. 4) Utilize a co-attention layer to compute the interaction relevance between substructures. 5) Predict DDI by aggregating the scores of substructure interactions. Next, Figure 1B shows the workflow of the soft mask adaptive graph neural network, which captures substructure information and readout graph representations through multi-layer sparse aggregation and weight convolution. The gradual change in node color represents the process of subgraph learning. More detailed illustration can be found in Section 3.3.

### 3.1 Problem definition

Given a set of drugs H , a set of interaction types $I={\left\{I_{i}\right\}}_{i=1}^{M}$ , and a dataset of DDIs $M={\left\{{\left(H_{x},H_{y},R\right)}_{i}\right\}}_{i=1}^{N}$ , where $H_{x}\in H$ and $H_{y}\in H$ denote a pair of drugs with interactions R of type $I_{i}$ , the DDI prediction task can be viewed as a solution function $f∶H\times J\times H\to \left[0,1\right]$ , which determines the probability that the combination of any two drugs may result in a given interaction type $I_{i}$ .

### 3.2 Input

For a given DDI tuple $\left(H_{x},H_{y},R\right)$ , this study takes the graph structure of drug pairs as input. Here, $H_{x}$ and $H_{y}$ , encoded as SMILES strings, are represented within a graph, denoted as $G=\left(V,E\right)$ . In graphs G , $V={\left\{u_{i}\right\}}_{i=1}^{n}$ is the set of nodes, node (vertices) u∈V represent atoms, $u_{i}$ denotes the *i*th atom, each atom $u_{i}$ has its corresponding eigenvector $h_{i}\subseteq R^{d}$ (d is the number of features), and edges $E={\left\{{\left(u_{a},u_{b}\right)}_{i}\right\}}_{i=1}^{m}\subset \left(V,V\right)$ denote the existence of a bond $\left(u_{a},u_{b}\right)$ between atoms $u_{a}$ and $u_{b}$ . Moreover, for each type of interaction, denoted as $I_{R}$ , which is a subset of all interactions I , we represent it through a learnable matrix, $M_{R}\in R^{D\times D}$ . The presence of an interaction R, between $H_{x}$ and $H_{y}$ is determined by applying this matrix $M_{R}$ .

### 3.3 Graph neural networks for adaptive substructure extraction

Traditional GNNs typically employ the strategy of neighborhood aggregation (or message passing) (Gilmer et al., 2017), which consists of three core modules: message passing module, neighborhood aggregation module, and prediction task module. These modules collaborate with each other to achieve effective modeling and prediction of graph-structured data. It iteratively updates the representation of each node by transforming and aggregating the representations of its neighboring nodes, and the aggregation operations of all nodes share the same parameters, with the structural information being implicitly learned. Specifically, each node collects messages from its neighboring nodes and updates its own feature representation through aggregation and combination functions. Finally, a readout function is used to integrate the node-level representations, aggregating the node features into a feature vector that represents the entire graph and is used for subsequent prediction tasks. However, in this process, the task-relevant structural information may be obscured by irrelevant (or noisy) parts, making it difficult to distinguish in downstream processing tasks, especially for long-range dependencies at higher layers in deep models (Li et al., 2018). To address this issue, soft-mask graph neural networks (Yang et al., 2021) adopt the idea of training all layers on the same subgraph, i.e., layer GNN is trained on a subgraph sequence of length, allowing for more flexible extraction of the desired subgraph through a masking mechanism. In this study, a variant of the soft mask graph neural network called the Multi-layer Adaptive Soft Mask Graph Neural Network (MASMG) was designed as the graph neural network for this study. MASMG aims to learn graph representations adaptively from the subgraph sequences of the original graph, with the purpose of capturing the substructure information of molecules and bypassing noise interference.

Considering that shallow convolutional layers cannot capture the global structure of molecules, MASMG layers are stacked to obtain substructure-level graph representations, as shown in Figure 1B. The substructure extraction part consists of k layers MASMG, and each graph convolutional layer includes sparse aggregation operations and weight convolution operations, updating the feature vector $h_{i}^{\left(k\right)}$ of node u in the ( k ) layer MASMG. The sparse aggregation operation propagates and aggregates information among nodes based on their edge relationships, capturing the local neighborhood information of nodes. The weight convolution operation weights and adjusts the features of nodes by learning the weight relationships between nodes, in order to better express the importance and interactions between nodes. The core idea of MASMG is to selectively extract nodes and edges using mask allocation, construct the desired subgraph, and perform feature propagation and aggregation within the subgraph scope. The soft mask is defined in continuous space to maintain different weights and differentiability. Compared with existing subgraph representation learning methods and graph pooling operations, the MASMG layer is not limited by fixed samples or dropout rates (Nyamabo et al., 2021), thus allowing for more flexible extraction of subgraphs of arbitrary sizes. In order to enable the MASMG layer to skip irrelevant parts and better capture the information of substructures, while focusing only on task-relevant substructures and learning representations of subgraphs with corresponding subgraph sequences, the expressive power and generalization ability of the model are improved. To this end, the formula for updating the corresponding embedding representation $h_{u}^{\left(k\right)}$ of the k layer is calculated as shown in Eq. 1.

$$h_{u}^{\left(k\right)}=\sigma \left(W^{\left(k\right)}m_{u}^{\left(k\right)}\left[h_{u}^{\left(k-1\right)}∥\sum_{s\in M\left(u\right)}m_{s}^{\left(k\right)}h_{s}^{\left(k-1\right)}\right]\right)$$ (1)

where σ denotes the ReLU activation function, ∥ represents the connection operation, $W^{\left(k\right)}\in R^{d\times d}$ is the matrix of trainable parameters, d denotes the dimension of the nodes, and $m_{u}^{\left(k\right)}\in \left[0,1\right]$ is the soft-mask of node u in the k -th layer. Specifically, $m_{u}^{\left(k\right)}$ is calculated as shown in Eq. 2.

$$m_{u}^{\left(k\right)}=\text{MLP}\left(\sigma \left(\left[I_{1}^{\left(k\right)}\left(m_{u}^{\left(k-1\right)}h_{u}^{\left(k-1\right)}\right)∥\sum_{s\in M\left(u\right)}I_{2}^{\left(k\right)}\left(m_{s}^{\left(k-1\right)}h_{s}^{\left(k-1\right)}\right)\right]\right)\right)$$ (2)

where $I_{1}^{\left(k\right)}$ and $I_{2}^{\left(k\right)}$ are linear mappings, σ is the ReLU activation function, and MLP is a 2-layer perceptron with an output feature dimension of 1. The last layer of the MLP is a Sigmoid activation function that determines the value of $m_{u}^{\left(k\right)}\in \left[0,1\right]$ on each node. $\sum_{s\in M\left(u\right)}I_{2}^{\left(k\right)}\left(m_{s}^{\left(k-1\right)}h_{s}^{\left(k-1\right)}\right)$ represents message passing, and the initial feature matrix for the first input is the feature matrix obtained through linear mappings.

After careful research, it has been determined that for the central node u , setting $m_{u}^{\left(k\right)}=0$ and we have $h_{u}^{\left(k\right)}=0$ . For the neighborhood $s\in M\left(u\right)$ of u , there is $h_{s}^{\left(k\right)}=\text{ReLU}\left(W^{\left(k\right)}m_{s}^{\left(k\right)}\left[h_{s}^{\left(k-1\right)}∥\sum_{s^{\prime }\in M\left(s\right)/\left\{v\right\}}m_{s^{\prime }}^{\left(k\right)}h_{s^{\prime }}^{\left(k-1\right)}\right]\right)$ , indicating that node u is not accessible to its neighbors. Therefore, for any subgraph $G_{S}$ of G , a layer of MASMG and a readout function can represent the subgraph $G_{S}$ , and other parts can be skipped.

The benefit of using soft-mask is that it takes into account the weights. The multiplication of $m_{u}^{\left(k\right)}$ and $h_{u}^{\left(k\right)}$ provides the weights of the aggregated operation’s substructure. As a result, it is possible to calculate the representation of the entire graph by utilizing the node representations read from different layers, and using SUM as the readout function. Here, we also use a jump cascade to generate a final graph-level representation $h_{x}^{\left(k\right)}$ of the drug $H_{x}\in H$ , as shown in Eq. 3.

$$h_{x}^{\left(k\right)}={\|}_{k=1}^{K}\left(\sum_{i=1}^{N}h_{i}^{\left(k\right)}\right)$$ (3)

### 3.4 Potential feature updates based on attention mechanism

After obtaining all the substructure information $h_{x}^{\left(k\right)}$ and $h_{y}^{\left(k\right)}$ (from the chemical substructure extraction part) of the input drugs $H_{x}$ and $H_{y}$ respectively from all MASMG layers, in order to simulate the correlation between drug substructure in both spatial and channel dimensions, this study utilizes an attention mechanism (Zhao et al., 2022) and applies MLP to transform them into attention vectors $ha_{x}$ and $ha_{y}$ , as shown in Eqs 4, 5. The purpose of this step is to separate feature extraction from attention modeling, converting the input features $h_{x}$ and $h_{y}$ into a more suitable representation for attention modeling to better handle the interaction between nodes.

$${ha_{x}}^{\left(i\right)}=\sigma \left(W_{h_{x}}∙h_{x}^{\left(i\right)}+b\right)$$ (4)

$${ha_{y}}^{\left(j\right)}=\sigma \left(W_{h_{y}}∙h_{y}^{\left(j\right)}+b\right)$$ (5)

where i=j=1,...,k , k represents the number of MASMG layers, σ is the non-linear activation function ReLU, $W_{h_{x}}$ and $W_{h_{y}}$ are weight matrices, and b is the bias vector. The attention vector $A_{x,y}$ is obtained by computing the correlation between vectors ${ha_{x}}^{\left(i\right)}$ and ${ha_{y}}^{\left(j\right)}$ as shown in Eq. 6, where $W_{a}$ is the weight matrix:

$$A_{x,y}=F\left(W_{a}∙\left({ha_{x}}^{\left(i\right)}+{ha_{y}}^{\left(j\right)}\right)+b\right)$$ (6)

After the aforementioned operations and by concatenating the attention vectors, the attention matrix $A_{x,y}$ is obtained, it contains interactions between drugs in both spatial and channel dimensions. This study generates the attention matrices $A_{h_{x}}$ and $A_{h_{y}}$ for the drugs by performing mean operations across different dimensions, as shown in Eqs 7, 8.

$$A_{h_{x}}=Sigmoid\left(Mean\left(A,2\right)\right)$$ (7)

$$A_{h_{y}}=Sigmoid\left(Mean\left(A,1\right)\right)$$ (8)

where Mean is the average operation that returns the average value of each row of the input in the given dimension. Sigmoid is the activation function that maps attention scores to the range (0, 1). The final representation ${\hat{h}}_{x}^{\left(i\right)}$ and ${\hat{h}}_{y}^{\left(j\right)}$ is obtained by completing the update of latent features:

$${\hat{h}}_{x}^{\left(i\right)}=h_{x}^{\left(i\right)}∙0.5+h_{x}^{\left(i\right)}⊗A_{h_{x}}$$ (9)

$${\hat{h}}_{y}^{\left(j\right)}=h_{y}^{\left(j\right)}∙0.5+h_{y}^{\left(j\right)}⊗A_{h_{y}}$$ (10)

where, in Eqs 9, 10, ⊗ represents element-wise multiplication.

### 3.5 Substructure interaction correlation

To explain the importance of pairwise interactions between drug substructures, after obtaining the final graph representation by updating latent features, this study further utilizes the common attention mechanism (Lu et al., 2016) to measure the importance $\gamma_{ij}$ of interactions between drug substructures ${\hat{h}}_{x}^{\left(i\right)}$ and ${\hat{h}}_{y}^{\left(j\right)}$ by calculating the attention weights. Here, i and j denote the substructures of drug x and drug y corresponding to the number of MASMG layers. The calculation is shown in Eq. 11.

$$\gamma_{ij}=z^{T}\tanh\left(W_{x}{\hat{h}}_{x}^{\left(i\right)}+W_{y}{\hat{h}}_{y}^{\left(j\right)}\right)$$ (11)

where, z is a learnable weight vector, and $W_{x}$ and $W_{y}$ are learnable weight matrices. By using different weight matrices, it avoids assigning higher scores to similar substructures, as this could complicate the entire learning process. This study also considers non-interacting drugs, which may receive negative scores, so tanh is used as the activation function to generate positive and negative outputs.

### 3.6 DDI prediction and loss function

In the DDI prediction task for the given DDI triplet $\left(H_{x},H_{y},R\right)$ , the probabilities are as follows:

$$P\left(H_{x},H_{y},R\right)=\sigma \left(\sum_{i=1}^{F}\sum_{j=1}^{F}\gamma_{ij}{\hat{h}}_{x}^{{\left(i\right)}^{T}}M_{R}{\hat{h}}_{y}^{\left(j\right)}\right)$$ (12)

where, σ represents the Sigmoid activation function, ${\hat{h}}_{x}^{\left(i\right)}$ and ${\hat{h}}_{y}^{\left(j\right)}$ are the updated latent feature embeddings of drugs $H_{x}$ and $H_{y}$ in k layer, $\gamma_{ij}$ is the attention score for the interaction between drug substructures, and $M_{R}$ is the trainable representation matrix for interaction type $I_{R}$ .

Therefore, for the DDI prediction task, it can be considered as a binary classification problem. In the given dataset, only known DDIs exist, and such triplets are regarded as positive samples indicating the presence of interactions. To generate negative samples, this study follows the negative sample generation strategy proposed by Wang et al. (2014), which involves randomly replacing $H_{x}$ or $H_{y}$ with a different drug. To balance the number of positive and negative samples, a 1:1 sampling ratio is commonly used, where the number of positive samples is equal to the number of negative samples. This setting helps to avoid the model’s excessive bias towards a particular class and maintains training balance. Additionally, this study employs the binary cross-entropy loss function to compare the prediction results of positive and negative samples, and the model parameters are updated through backpropagation and gradient descent algorithms for end-to-end training, with the loss function as shown in Eq. 13.

$$L=-\frac{1}{\left|N\right|}\sum_{l\in N}\left(\log\left(p_{l}\right)+\log\left(1-p_{l}^{\prime }\right)\right)$$ (13)

where, $p_{l}$ represents the probability of positive samples, $p_{l}^{\prime }$ represents the probability of corresponding negative samples, $\left|N\right|$ represents the number of DDI triplets in the dataset, and $l=\left(H_{x},H_{y},R\right)$ represents a DDI triplet. The probabilities of samples $p_{l}$ and $p_{l}^{\prime }$ are calculated by the scoring function defined in Eq. 12.

## 4 Experiments

In this section, we evaluate our MASMDDI method by multiple experiments. Specifically, Section 4.1, Section 4.2 and Section 4.3 first describe the two real datasets used in the experiments and the experimental setup, as well as the experimental training and evaluation metrics. Next, Section 4.4 and Section 4.5 introduce the baseline and experimental results in detail. Then, Section 4.6 and Section 4.7 show the ablation experiments and parameter sensitivity analysis. At last, Section 4.8 and Section 4.9 provide the visual analysis of the relationships and the model efficiency analysis.

### 4.1 Dataset

This research employs two widely used datasets, DrugBank (Wishart et al., 2018) and Twosides (Tatonetti et al., 2012), to evaluate the model MASMDDI.

DrugBank is a unique bioinformatics and chemoinformatics resource that integrates comprehensive drug data with detailed drug-target information (Wishart et al., 2018). It contains 191,808 DDI tuples, 1,706 drugs, and 86 types of interactions. Within the DrugBank dataset, each drug pair is associated with a single type of interaction, describing how one drug affects the metabolism of another drug. Typically, given two drugs with SMILES sequences, the ultimate goal is to predict their interaction types (i.e., binary, multiclass, and multilabel classification).

Twosides was constructed by Zitnik et al. (2018) through preprocessing and filtering of the original Twosides dataset. The Twosides dataset collects polypharmacy side effects associated with drug pairs or individual drugs in higher-order drug combinations. It includes 645 drugs, 963 interaction types, and 4,576,287 DDI tuples. In contrast to the DrugBank dataset, the Twosides dataset encompasses multiple interaction types for drug pairs. According to Zitnik et al. (2018), interaction types with fewer than 500 DDI tuples were removed, and further preprocessing retained only the most common types. Therefore, the final dataset contains 963 interaction types and 4,576,287 tuples.

### 4.2 Experimental setup

To assess the generalization ability of our model, experiments on the DrugBank dataset are divided into transductive and inductive scenarios. The transductive scenario, often referred to as the warm-start scenario, is the most common dataset split scheme. During the transductive scenario, the dataset is randomly split into a training set (60%), a validation set (20%), and a test set (20%). It is noteworthy that the drug entities used during training, validation, and testing are consistent; in other words, the model does not encounter new drugs during the testing phase that it has not seen during training. This design avoids the “cold start” problem that the model might face in practical applications, where it has to predict interactions between new drugs without prior knowledge.

The inductive scenario is more challenging than the transductive scenario. In this scenario, the test set includes drugs that either partially or entirely lack representation in the training set, resembling real-world scenarios where there may be new drugs with unknown interactions. In such a cold-start scenario, the model requires strong generalization capabilities as it lacks prior knowledge of the unseen drugs during the training process. To create this scenario, this study randomly selects 20% of the drugs as unknown drugs, with the remaining 80% being known drugs. The rest of the experimental setup is the same as in the warm-start scenario. It is essential to note that in the cold start scenario, experiments are conducted exclusively within the DrugBank dataset. This is due to the presence of false positives in the Twosides dataset, meaning that it contains drug pairs that do not actually interact in practice, which would lead to unreliable evaluations in the cold-start scenario. To prevent the models from overfitting to the drugs in the training data in the cold-start scenario, a weight decay of 0.0005 is applied to all methods. This approach helps reduce the model’s tendency to overfit and improves its generalization performance in cold-start scenarios.

Specifically, this study employs two partitioning schemes to construct test sets:

**S1:** In the S1 test set, each DDI sample has two unknown drugs in the training set. The task is to predict DDIs for a pair of new drugs for which there are no existing interactions with any drugs in the training set.

**S2:** In the S2 test set, each DDI sample has one known drug and one unknown drug in the training set. The primary task is to predict DDIs for a new drug when combined with another existing drug for which no interactions are known.

### 4.3 Model training and evaluation indicators

MASMDDI comprises k = 4 layers of MASMG modules, with each module consisting of four sparse aggregation layers and weighted convolutional layers (see Figure 1). To be specific, the RDKit (Bento et al., 2020) package converts these strings into a graph structure, and the molecular graph structures are passed through the MASMDDI layer to generate hidden feature representations of size 128 as the final output. In this case, each drug node receives a raw chemical feature input of dimension 55, the input data is normalized using the LayerNorm layer, and each interaction type $I_{R}\subseteq I$ is represented by a learnable matrix $M_{R}$ of dimension 128. In particular, a threshold of 0.5 is utilized to distinguish predictions in this study: those with a probability exceeding 0.5 are considered positive 1), whilst all other predictions are identified as negative 0). Finally, The study employed Python (Paszke et al., 2019) and Pytorch Geometrics (Fey and Lenssen, 2019) for model training. The experiments are conducted on a server equipped with a Tesla V100-32 GB GPU. The model was trained on a small batch of 128 tuples of DDIs, utilizing the Adam SGD optimizer (Kingma and Ba, 2014). The optimization algorithm employed a learning rate $l_{r}=5e-4\times 0.96t$ with an exponential decay schedule, where t corresponds to the current epoch, and a weight decay with a set value of 5e−4 was utilized to counteract overfitting. To assess performance, four evaluation metrics are employed in this study: accuracy (ACC), area under the subject operating characteristic curve (AUROC), area under the accuracy-recall curve (AUPRC), and F1 scores. In all cases, higher values indicate superior performance.

### 4.4 Baselines

In this study, MASMDDI was compared with several state-of-the-art DDI baseline methods in a transductive and inductive setting, including MHCADDI (Andreea et al., 2019), GAT-DDI (Velickovic et al., 2018; Nyamabo et al., 2022), MR-GNN (Xu et al., 2019), SSI-DDI (Nyamabo et al., 2021), GMPNN-CS (Nyamabo et al., 2022) and SA-DDI_GMP (Yang et al., 2022). All these methods are chemical structure-based methods. Among them, MR-GNN and MHCADDI are molecular map dependent methods that consider the entire chemical structure of the drug. MASMDDI, SSI-DDI, GAT-DDI, GMPNN-CS and SA-DDI_GMP are substructure-based GNN methods. Different from the other methods, MASMDDI uses a soft-mask GNN. This enables more flexibility in extracting subgraphs of arbitrary sizes compared to existing subgraph representation learning methods and graph pooling operations, which are limited by fixed samples or discard rates.

• MR-GNN (Xu et al., 2019): effectively captures the complex interactions between entities in the knowledge graph by combining multi-resolution modeling and dual graph neural networks for DDI prediction tasks.
• MHCADDI (Andreea et al., 2019): constructs a drug interaction network and introduces a graph co-attention mechanism to capture the interrelationships between drugs.
• SSI-DDI (Nyamabo et al., 2021): utilizes substructure features to represent the characteristics of drug molecules and predicts DDI by calculating substructure interactions.
• GAT-DDI (Velickovic et al., 2018; Nyamabo et al., 2022): directly utilizes Graph Attention Networks (GAT) for drug modeling and DDI prediction.
• GMPNN-CS (Nyamabo et al., 2022): A Gated Message-Passing Neural Network (GMPNN) was designed to learn chemical substructures of different sizes from molecular graphical representations of drugs for predicting DDIs between drugs.
• SA-DDI_GMP (Yang et al., 2022): The method was proposed by Yang et al. (2022) The model uses a substructure-aware graph neural network with an attention mechanism to extract adaptive substructures for DDI prediction.

### 4.5 Results

**FIGURE 2:**
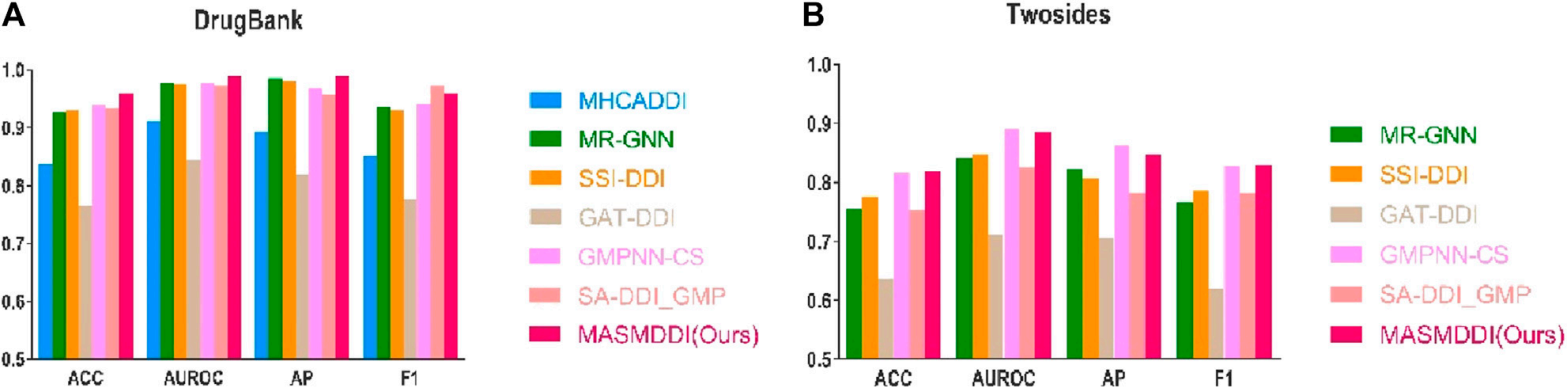
Experimental Performance of MASMDDI and Baseline Models in transductive setting. **(A)** DrugBank dataset. **(B)** Twosides dataset.

Table 1 displays the predictive performance of MASMDDI compared to previous models on DrugBank and Twosides datasets in transductive setting. In the transduction setup, drugs used for training may also be present in the test set. To ensure unbiased evaluation, datasets are randomly split based on the DDI tuple. We split both datasets hierarchically by interaction type to maintain the same proportion of interaction types in the training (60%), validation (20%), and test (20%) sets, and for each DDI tuple, a negative sample is generated. Due to limited computational resources, we could not run the MHCADDI model on the Twosides dataset, so we only reference the AUROC results from the original paper. Despite the already high accuracy achieved by existing methods in DDI prediction, our model demonstrates further breakthroughs in performance. On the DrugBank dataset, except for the F1 scores, MASMDDI outperformed the other models on the other three evaluation metrics, with ACC and AUROC reaching 0.9596 and 0.9903, respectively. In comparison to the SSI-DDI model, it shows a 2.93% improvement in ACC and a 3.03% improvement in F1 scores. This indicates that MASMDDI, in a direct-impact setting, can effectively distinguish interacting drugs from non-interacting drugs and address the prediction of existing drug DDIs with high precision. MASMDDI also performs exceptionally well on the Twosides dataset, achieving a score of 0.8183 for ACC, and AUPRC and F1 scores of 0.8472 and 0.8288, respectively. These experimental results validate the effectiveness of the DDI prediction method proposed in this paper. To provide a more intuitive representation of the experimental results, Figure 2 illustrates bar graphs of MASMDDI and other models for these four-evaluation metrics on the two datasets (MHCADDI is not shown in the Twosides bar graph due to missing data).

**TABLE 1:** Experimental results of MASMDDI and baseline in transductive setting on DrugBank and Twosides.

Method	DrugBank				Twosides			
	ACC	AUROC	AUPRC	F1	ACC	AUROC	AUPRC	F1
MR-GNN	0.9283	0.9785	0.9862	0.9367	0.7562	0.8402	0.8225	0.7663
MHCADDI	0.8380	0.9116	0.8926	0.8506	-	0.8820	-	-
SSI-DDI	0.9303	0.9758	0.9809	0.9304	0.7766	0.8481	0.8077	0.7856
GAT-DDI	0.7652	0.8437	0.8186	0.7760	0.6363	0.7113	0.7059	0.6199
GMPNN-CS	0.9396	0.9777	0.9696	0.9407	0.8153	**0.8899**	**0.8625**	0.8274
SA-DDI**_**GMP	0.9354	0.9722	0.9580	**0.9722**	0.7532	0.8259	0.7822	0.7814
**MASMDDI**	**0.9596**	**0.9903**	**0.9894**	0.9601	**0.8185**	0.8855	0.8475	**0.8291**

Bold vales indicate optimal effects.

Table 2 summarizes the experimental results in two tasks under the inductive scenario. In the inductive scenario, this study chose to conduct experiments using only the DrugBank dataset. This is because DrugBank contains a richer number of drugs compared to the Twosides dataset. If the number of drugs in the selected dataset is too small, it will result in the model overfitting the features of the old drugs in the inductive scenario. To prevent overfitting, this study also added a discard layer to MASMDDI. From the results, it can be observed that the predictive performance of MASMDDI decreases significantly in the inductive scenario compared to the transductive scenario. This indicates that the lack of prior knowledge about chemical structure information reduces the model’s generalization ability and highlights the challenge faced by DDI prediction models in improving their generalization ability. Despite the overall decrease in model performance, the performance of MASMDDI in the S2 task is excellent, especially for the high index of the F1 score. This indicates that the model’s classification prediction performance is better in the S2 task, while worse in the S1 task. This is due to the significant differences in the core chemical structures of most drugs in the DrugBank dataset and data imbalance. These factors are important to be considered in future work. In practical applications, the combination therapy of one unknown drug and one known drug in S2 task is more common and safe compared to the combination therapy of two unknown drugs in S1 task. Therefore, the excellent performance of MASMDDI in task S2 demonstrates the potential practical value of the proposed method in real-world treatments.

**TABLE 2:** Experimental results of MASMDDI and baseline in inductive setting on the DrugBank.

Method	S1 (new drug - new drug)				S2 (new drug - old drug)			
	ACC	AUROC	AUPRC	F1	ACC	AUROC	AUPRC	F1
MR-GNN	0.6192	0.6689	0.6431	0.6071	0.6733	0.7652	0.7525	0.5971
MHCADDI	0.6650	0.7253	0.7106	**0.6721**	0.7058	0.7784	0.7616	0.7274
SSI-DDI	0.5964	0.6937	0.7118	0.3854	0.6948	0.7816	0.7967	0.6170
GAT-DDI	0.6340	0.6968	0.7038	0.5952	0.6409	0.7075	0.7060	0.6284
GMPNN-CS	**0.6863**	**0.7472**	**0.7453**	0.6638	0.7866	0.8666	0.8469	0.7966
SA-DDI**_**GMP	0.6355	0.6888	0.6609	0.6460	**0.7939**	**0.8812**	**0.8773**	0.7646
**MASMDDI**	0.6141	0.7014	0.7407	0.4680	0.7260	0.8014	0.8086	**0.8576**

Bold vales indicate optimal effects.

### 4.6 Ablation study

To assess the influence of the multi-layer MASMG layers and substructure attention mechanism proposed in this study and to gauge the effectiveness of the MASMDDI method, disintegration experiments were conducted by altering the combination of different numbers of MASMG layers and the presence of the substructure attention mechanism module in the model. Each experiment was run for 100 epochs. Experiments were performed on DrugBank for models with 3, 4, and 5 layers, and on Twosides for models with 2, 3, and 4 layers.

Table 3 and Table 4 show the results illustrating how the number of layers impacts the performance of the MASMG model. In both tables, “L#-N/A” represents experiments conducted without using the substructure attention mechanism but with # layers of MASMG layers (e.g., “L3-N/A” means using 3 MASMG layers without adding the substructure attention module), and “L&-A” represents the complete experiments conducted with both # layers of MASMG and the substructure attention mechanism. From Table 3 and Table 4, it can be observed that the model with 4 MASMG layers and the substructure attention module in DrugBank achieves the best predictive performance, while the best performance was achieved by the model with 3 MASMG layers and substructure focus modules in Twosides.

**TABLE 3:** MASMDDI ablation experiment results. “L#-N/A” represents experiments conducted without using the substructure attention mechanism but with # layers of MASMG layers (e.g., “L3-N/A” means using 3 MASMG layers without adding the substructure attention module), and “L#-A” represents the complete experiments conducted with both # layers of MASMG and the substructure attention mechanism.

Method	ACC	AUROC	AP	F1
MASMDDI_L3-N/A	0.8929	0.9496	0.9380	0.8940
MASMDDI_L4-N/A	0.9203	0.9688	0.9619	0.9219
MASMDDI_L5-N/A	0.9338	0.9785	0.9735	0.9379
MASMDDI_L3-A	0.9527	0.9890	0.9879	0.9573
**MASMDDI_L4-A**	**0.9596**	**0.9903**	**0.9894**	**0.9601**
MASMDDI_L5-A	0.9542	0.9881	0.9867	0.9548

Bold vales indicate optimal effects.

**TABLE 4:** MASMDDI ablation experiment results. “L#-N/A” represents experiments conducted without using the substructure attention mechanism but with # layers of MASMG layers (e.g., “L3-N/A” means using 3 MASMG layers without adding the substructure attention module), and “L#-A” represents the complete experiments conducted with both # layers of MASMG and the substructure attention mechanism.

Method	ACC	AUROC	AP	F1
MASMDDI_L2-N/A	0.7804	0.8458	0.8010	0.7929
MASMDDI_L3-N/A	0.8023	0.8684	0.8254	0.8143
MASMDDI_L4-N/A	0.8094	0.8772	0.8374	0.8196
MASMDDI_L2-A	0.8100	0.8784	0.8407	0.8206
**MASMDDI_L3-A**	**0.8185**	**0.8855**	**0.8475**	**0.8291**
MASMDDI_L4-A	0.8156	0.8823	0.8431	0.8262

Bold vales indicate optimal effects.

**FIGURE 3:**
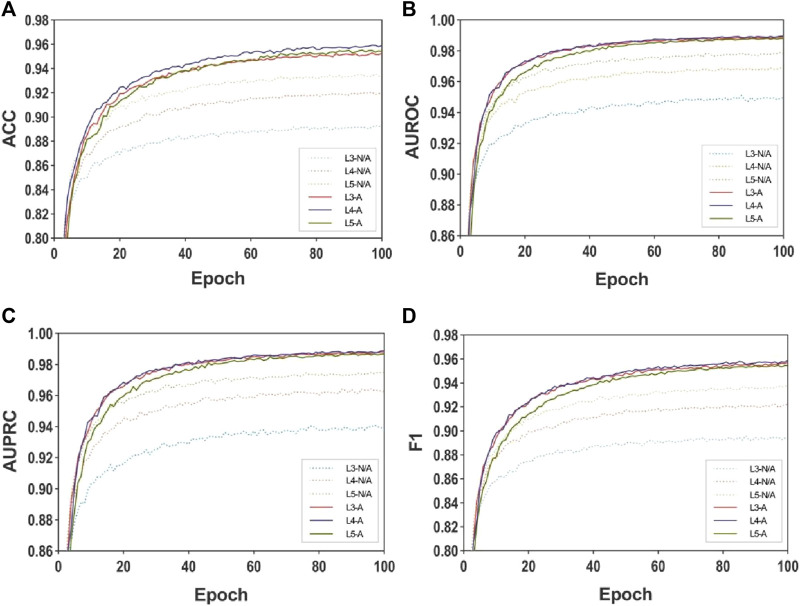
Ablation experiments of MASMDDI with respect to substructure attention mechanism on the DrugBank dataset. Panels represent performance results using 3 to 5 layers of MASMG modules and/or adding substructure attention mechanisms, where **(A)** ACC, **(B)** AUROC, **(C)** AUPRC, **(D)** F1.

**FIGURE 4:**
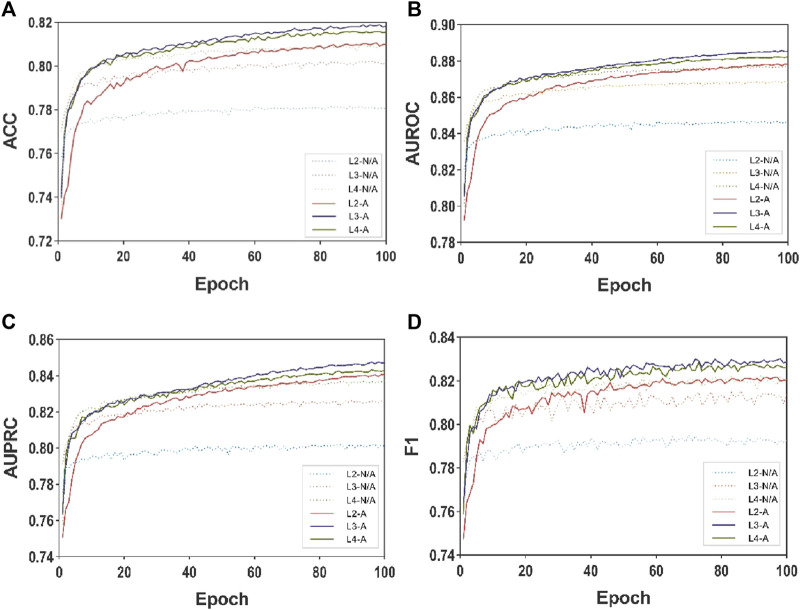
Ablation experiments of MASMDDI with respect to substructure attention mechanism on the Twosides dataset. Panels represent performance results using 3 to 5 layers of MASMG modules and/or adding substructure attention mechanisms, where **(A)** ACC **(B)** AUROC, **(C)** AUPRC, **(D)** F1.

Figure 3 and Figure 4 presents the line plots of (A)ACC, (B)AUROC, (C)AUPRC and (D)F1 results obtained from the ablation study of MASMDDI on the DrugBank and Twosides dataset in the transductive setting. The models using the substructure attention module outperform the models without it in all the evaluation metrics. It can be observed that the substructure attention module plays a crucial role, as the performance of the shallow model (L3-A) surpasses that of the deep model without the substructure attention module (L5-N/A) and similarly the L2-A outperforms the L4-N/A in Twosides. This indicates that the substructure attention module effectively enhances the latent feature representation of substructure features extracted by the MASMG layers in both datasets.

### 4.7 Parameter analysis experiment

In this section, we will comprehensively assess the reasonableness of the model in terms of both the number of MASMG layers and the impact of batch and dimension on model performance.

#### 4.7.1 Analysis of layers in adaptive soft mask graph neural networks

**FIGURE 5:**
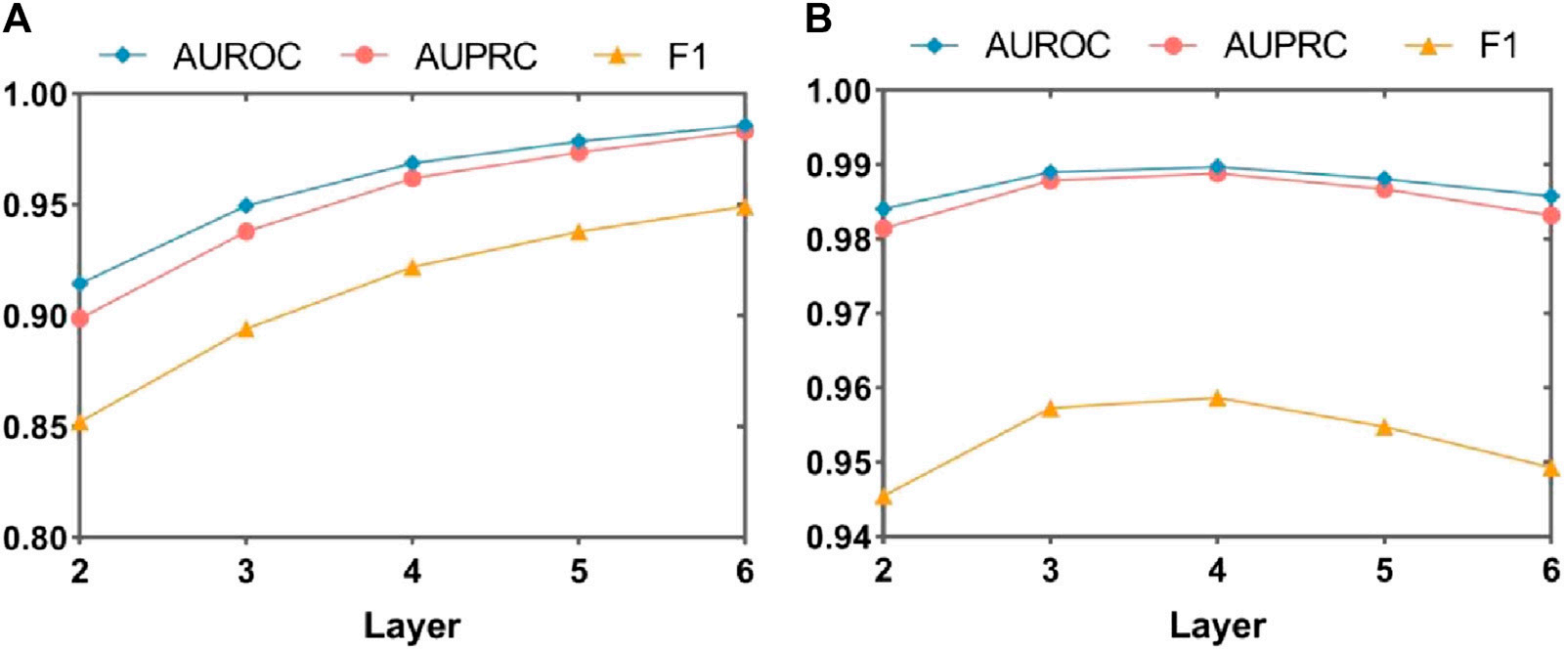
Experimental study on the effect of MASMG layers on the performance of MASMDDI model on DrugBank dataset. Panel **(A)** shows the effect of increasing or decreasing the number of MASMG layers on model performance in the case of removing structural attention mechanisms, and panel **(B)** shows the effect of increasing or decreasing the number of MASMG layers on model performance in the case of adding structural attention mechanisms.

**FIGURE 6:**
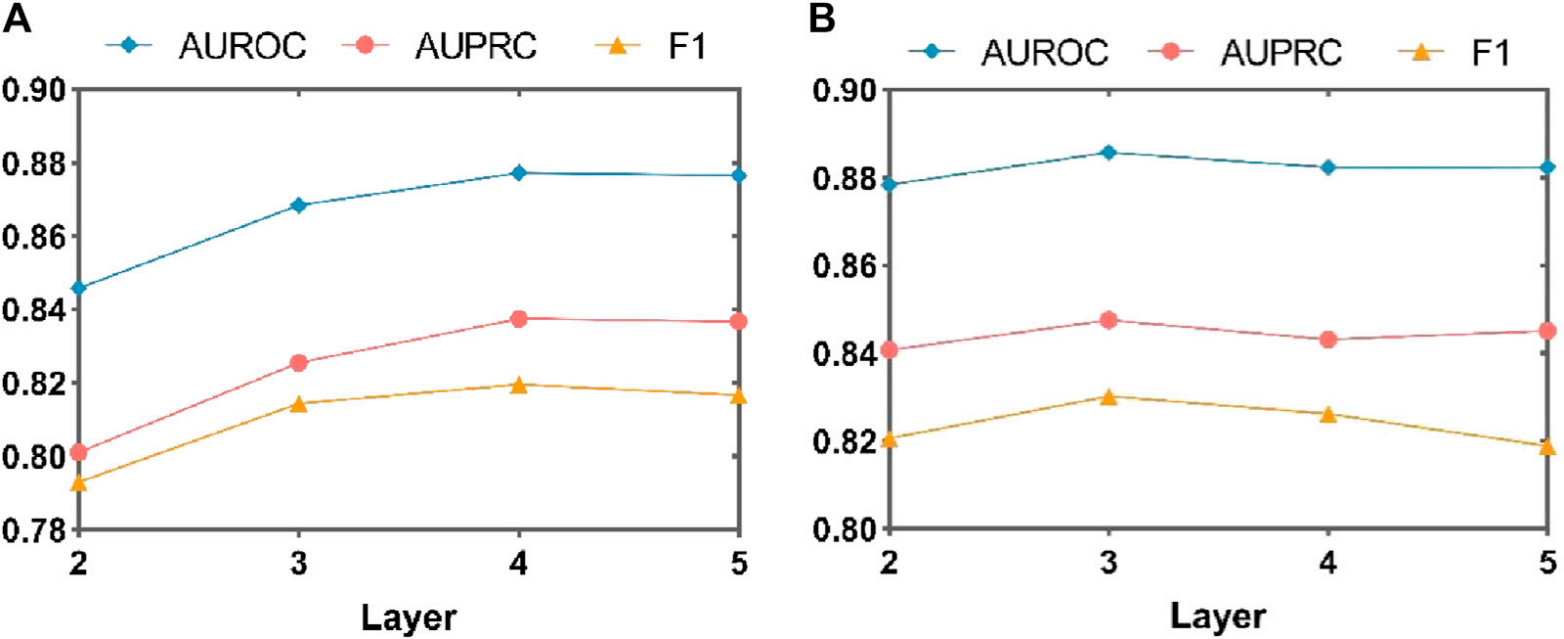
Experimental study on the effect of MASMG layers on the performance of MASMDDI model on Twosides dataset. Panel **(A)** shows the effect of increasing or decreasing the number of MASMG layers on model performance in the case of removing structural attention mechanisms, and panel **(B)** shows the effect of increasing or decreasing the number of MASMG layers on model performance in the case of adding structural attention mechanisms.

To comprehensively evaluate the impact of the number of layers in the MASMG on model performance, this study conducted experiments by removing the substructure attention module and/or increasing or decreasing the number of MASMG layers to adjust the model’s depth. MASMG with 2–6 and 2 to 5 layers were selected for experimentation in DrugBank and Twosides, respectively, and each setting was trained for 100 epochs. Figure 5 and Figure 6 illustrates the results using a dot-line graph format for AUROC, AUPRC, and F1 scores. Figure 5A and Figure 6A displays the experimental results of the MASMG models using only multiple layers. These results indicate that as the number of MASMG layers increases, the model’s performance also improves. However, the performance improvement gradually diminishes, suggesting a diminishing effect of depth for the MASMG. To gain a more comprehensive understanding of model performance, we further considered the case of using both multiple layers of MASMG and the substructure attention module in Figure 5B and Figure 6B. It is evident that the model achieves the best performance in DrugBank when the number of MASMG layers is 4, and its performance is best in Twosides when the number of MASMG layers is 3. Excessive layers lead to a decrease in performance, indicating that overly deep layers result in overfitting or gradient vanishing issues.

#### 4.7.2 Hyperparameter batch and dim analysis

**FIGURE 7:**
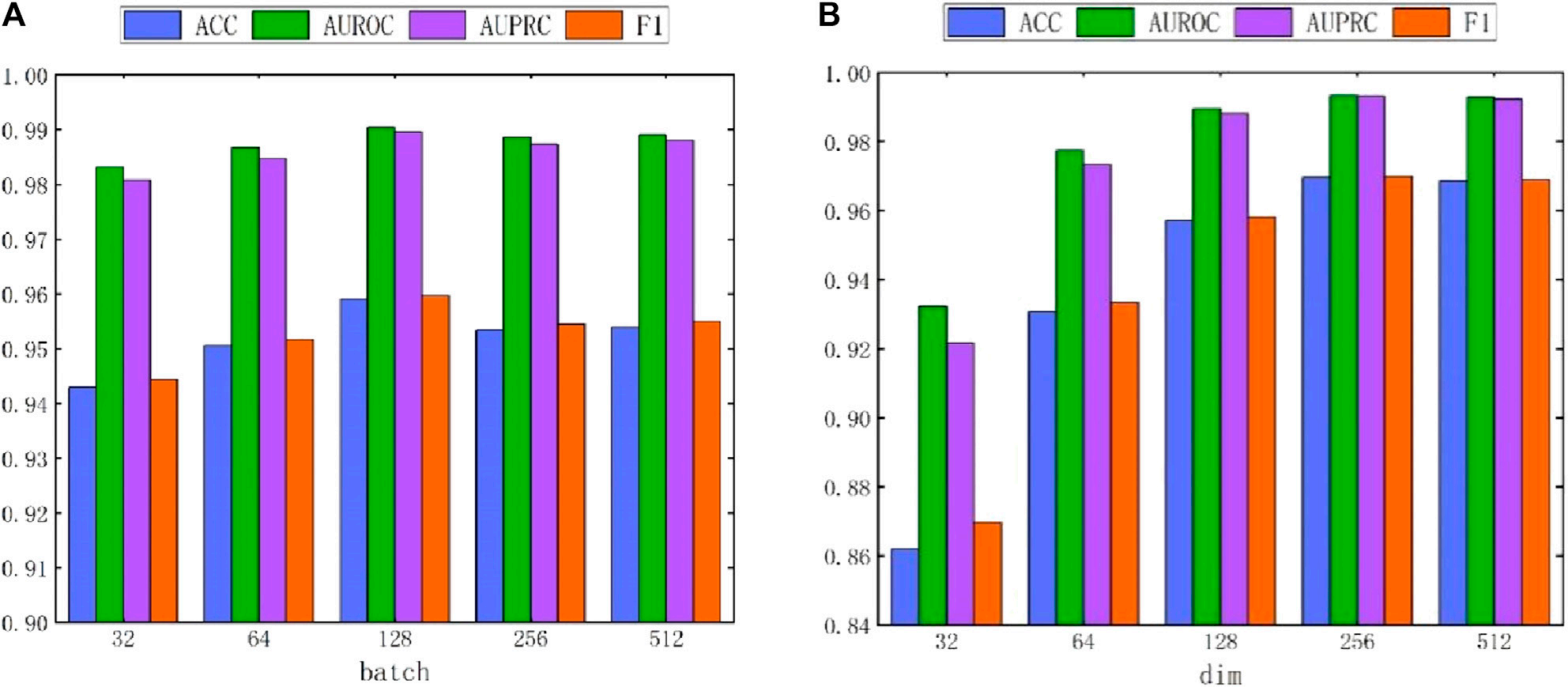
The performance impact of batch and hidden dimension dim on the MASMDDI model in DrugBank dataset. **(A)** batch. **(B)** dim.

**FIGURE 8:**
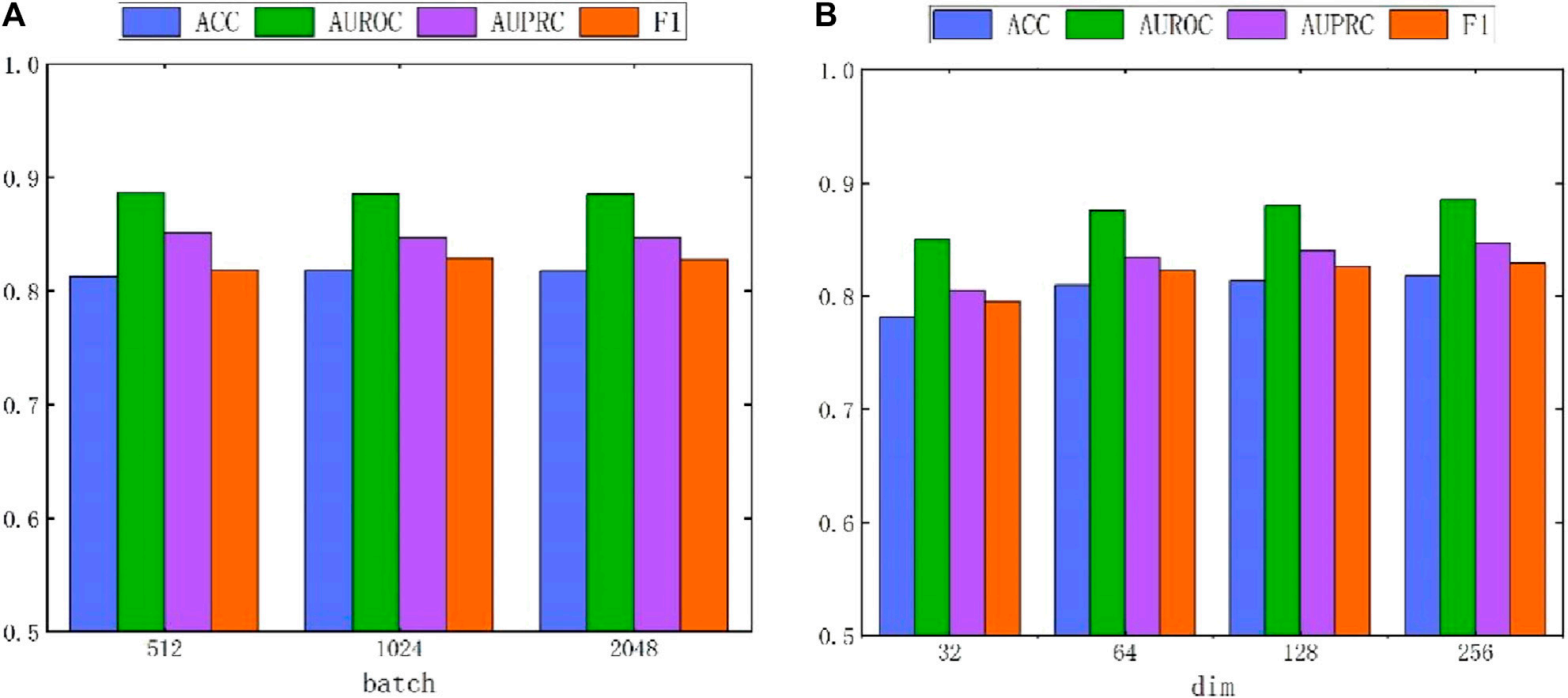
The performance impact of batch and hidden dimension dim on the MASMDDI model in Twosides dataset. **(A)** batch. **(B)** dim.

Due to the adoption of batch sampling and training strategies in MASMDDI, the batch size is particularly important. This section delves into the impact of different batch sizes on the method’s performance. Notably, setting the batch size too small can hinder the model’s ability to converge effectively. Conversely, if the batch size is set too large, the computational cost will significantly increase. Both situations can lead to a decrease in model performance. Therefore, we conducted in-depth research on the impact of different batch sizes on method performance. As shown in Figure 7A, when the batch size is set to 128, the method exhibits the best performance in DrugBank. As depicted in Figure 8A, the influence of batch size on model performance in Twosides is relatively minor. Optimal performance is observed when the batch size is set to 1024.

In addition, this study also conducted experiments on the impact of hidden dimensions on model performance. As shown in Figure 7B and Figure 8B, the larger the hidden dimension of the model, the better its performance. However, as the dimension increases, the performance improvement gradually diminishes, and performance tends to stabilize when the hidden dimension reaches 256. Experimental results for these two parameters indicate that when the hidden dimension is set to 256 and the batch size is set to 128, MASMDDI achieves significantly improved performance compared to other settings on DrugBank. When the hidden dimension is set to 256 and the batch size is set to 1024, MASMDDI has better performance on Twosides. These series of experimental results not only emphasize the criticality of batch size and hidden dimension but also provide strong support for selecting optimal hyperparameters. These findings provide valuable guidance for further optimizing the performance of MASMDDI. Future research endeavors may delve deeper into refining these strategies to enhance not only the efficiency but also the scalability of MASMDDI in handling larger datasets and more complex learning scenarios.

### 4.8 Visual analysis of relationships

**FIGURE 9:**
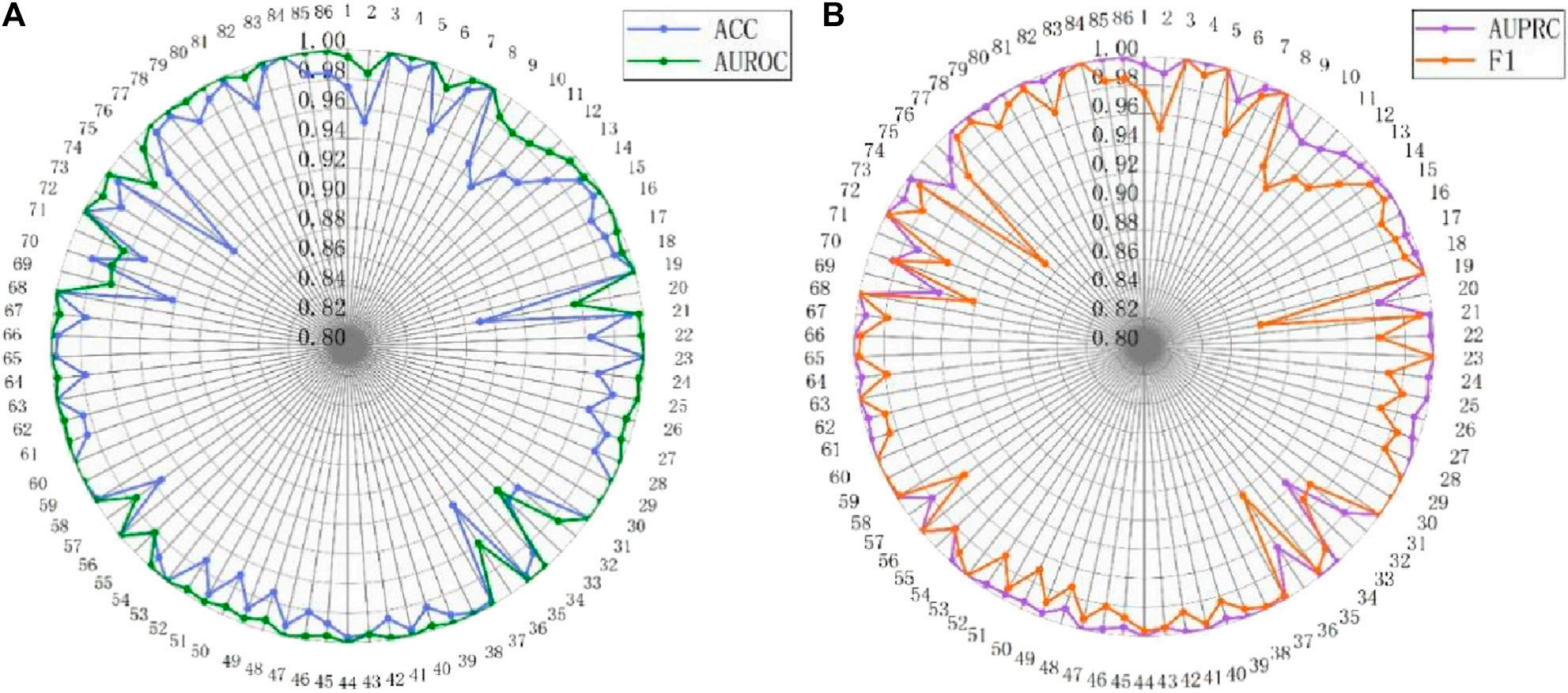
Performance radar chart for each DDI type on DrugBank. **(A)** ACC and AUROC. **(B)** AUPRC and F1.

**FIGURE 10:**
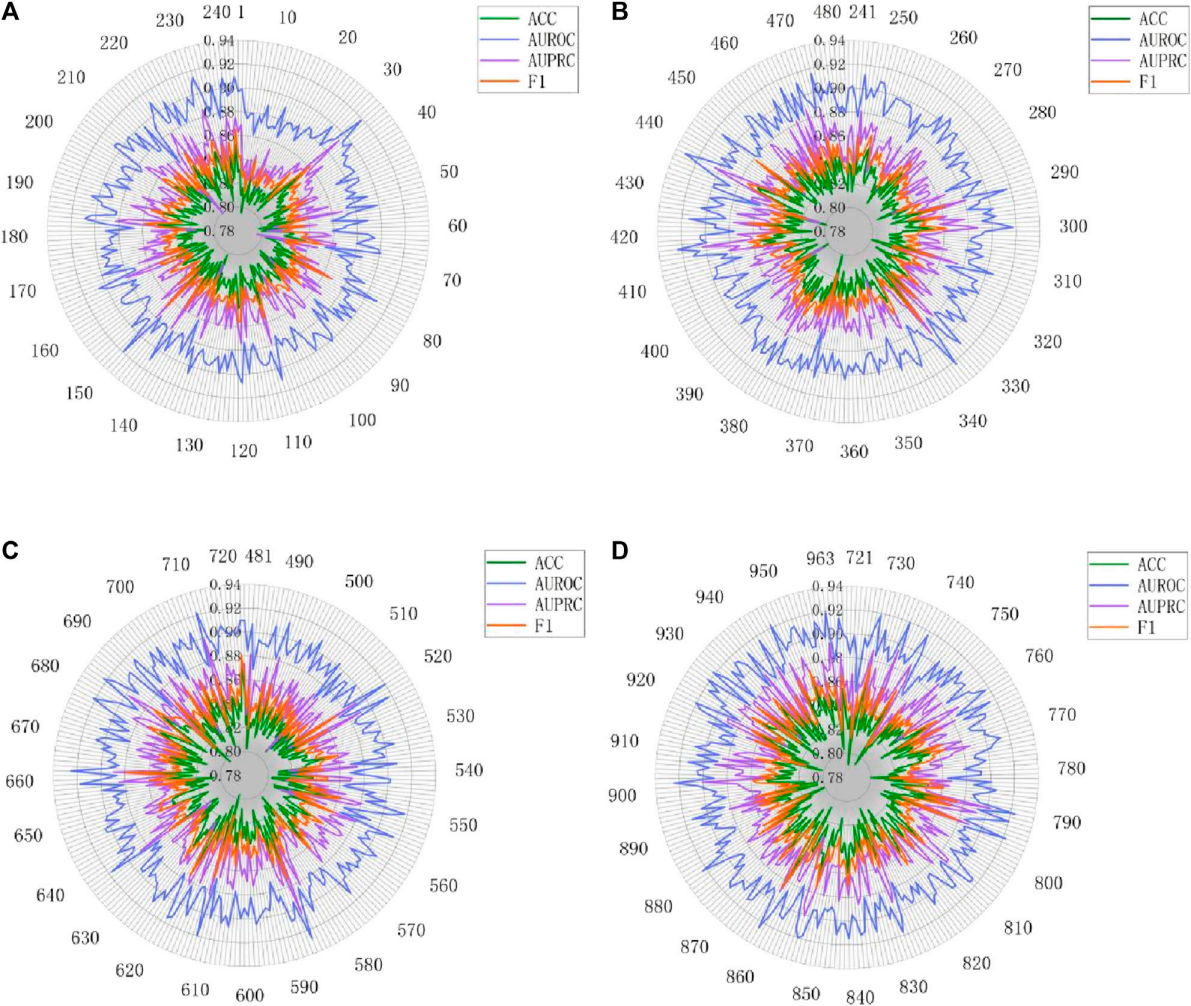
Performance radar chart for each DDI type on Twosides. **(A)** DDI type 1-240. **(B)** DDI type 241-480. **(C)** DDI type 481-720. **(D)** DDI type 720-963.

The analysis of batch size and hidden dimension demonstrates the outstanding performance of MASMDDI in overall performance. To gain a deeper understanding of the model’s effectiveness, this study further conducted extensive experimental predictions on the performance of the model for each type of DDIs on the DrugBank and Twosides dataset. Evaluation metrics for each interaction type were independently calculated using the predicted scores and true labels. The detailed presentation of these performance metrics can be seen in Figures 9, 10. Analysis of Figure 9 reveals that out of the 86 DDI types examined on the DrugBank dataset, MASMDDI achieved the highest AUROC scores and highest AUPRC scores (over 88%) for 82 DDI types. This indicates that MASMDDI has strong generalization ability and prediction accuracy for multiple types of drug interactions. However, we observed significant variations in accuracy for each relationship when studying specific relationships. For example, the accuracy for type 75 was relatively low, while the accuracy for type 59 was much higher.

To further explore this phenomenon, we carefully examined the number of samples for each relationship and found that some relationship types had relatively few samples. For example, type 75 had only 20 samples available for prediction. This imbalanced distribution of samples may affect the model’s performance on specific relationships. However, analysis of Figure 10 shows that the four metrics of MASMDDI among the 963 DDI types on the Twosides dataset are relatively average, with scores around or above 0.8. This shows that the Twosides dataset is a relatively balanced dataset. In this study, we also concluded that the prediction of DDIs actually involves an issue of relationship imbalance. This means that there are significant differences in the sample sizes for each relationship, and the imbalance in data distribution is a key issue that needs to be addressed in future research. This in-depth analysis not only highlights the strong performance of the MASMDDI model but also provides valuable insights for future related research. It encourages researchers to pay more attention to the challenge of relationship imbalance and seek solutions to improve the robustness of the models.

### 4.9 Model efficiency analysis

In assessing the performance of models on the DrugBank and Twosides datasets, the comparison of time and memory efficiency is also a crucial metric, directly impacting the feasibility and scalability of the model in practical applications. Due to the varying sizes and complexities of the two datasets, they affect time and memory efficiency differently. Twosides, the larger dataset, requires more memory for storage and takes longer processing time. We compare the efficiency of MASMDDI with the best baseline GMPNN-CS, and Table 5 presents the average computation time and maximum memory usage of MASMDDI and GMPNN-CS on the DrugBank and Twosides datasets, indicating that MASMDDI consumes less computation time on both datasets compared to GMPNN-CS. However, GMPNN-CS exhibits relatively lower memory usage on the Twosides dataset due to its optimized algorithm tailored for this dataset. From this experiment, we can conclude that for future research endeavors aimed at enhancing time and memory efficiency, techniques such as algorithm optimization and parallel computing can be employed.

**TABLE 5:** Comparison of time and memory efficiency on DrugBank and Twosides datasets.

Method	DrugBank		Twosides	
	Time (s)	Memory (G)	Time (s)	Memory (G)
GMPNN-CS	97.70	2.90	2041.25	**3.65**
**MASMDDI**	**73.89**	**1.80**	**1817.97**	6.47

Bold vales indicate optimal effects.

## 5 Conclusion

This study introduces a computational method MASMDDI using a soft mask adaptive graph neural network to predict DDIs. The excellent performance of MASMDDI is mainly attributed to the recognition that DDIs are fundamentally driven by interactions between chemical substructures. The model introduces multi-layered soft mask GNN and substructure attention mechanisms to flexibly learn drug substructures of different sizes and shapes and establish their interaction models, thereby inferring potential DDIs based on chemical compositions. In experiments, we evaluate the performance of MASMDDI using two real-world datasets, DrugBank and Twosides. The results show that MASMDDI outperforms the baseline in transductive setting and is competitive in the inductive setting. However, MASMDDI still has limitations. Even though MASMDDI performs superiorly on both datasets in transduction scenarios, it is predictive performance for DDIs of two new drugs is slightly lower in induction scenarios. Moreover, the time consumption and model complexity are relatively high, leading to slightly higher time costs. Future research can focus on improving the model’s generalization ability in the inductive learning paradigm while enhancing its applicability. Enhancing MASMDDI’s ability to infer knowledge from limited or unseen drug interactions can significantly improve its practicality and relevance in the dynamic environment of pharmaceutical research and drug development. Possible approaches include adopting graph contrastive learning methods or balancing datasets.

## Data Availability

All datasets and codes covered in the study are available at https://github.com/linjunpeng1998/MASMDDI.

## Abbreviations

ACC, Accuracy; AUROC, Area under the receiver operating characteristic curve; AUPRC, Area under the precision-recall curve; DDI, Drug‒drug interaction; MASMG, Multi-layer adaptive soft mask graph neural network; MLP, Multi-layer perceptron.
